# Factors Associated with Adherence to the Mediterranean Diet among Adolescents Living in Sicily, Southern Italy

**DOI:** 10.3390/nu5124908

**Published:** 2013-12-04

**Authors:** Giuseppe Grosso, Stefano Marventano, Silvio Buscemi, Alessandro Scuderi, Margherita Matalone, Alessio Platania, Gabriele Giorgianni, Stefania Rametta, Francesca Nolfo, Fabio Galvano, Antonio Mistretta

**Affiliations:** 1 Department “G.F. Ingrassia”, Section of Hygiene and Public Health, University of Catania, Catania 95123, Italy; E-Mails: stefano.marventano@studium.unict.it (S.M.); alessioplatania@me.com (A.P.); giorgiangab@gmail.com (G.G.); steframetta@gmail.com (S.R.); fra.nolfo@gmail.com (F.N.); anmist@unict.it (A.M.); 2 Department of Clinical and Molecular Biomedicine, Section of Pharmacology and Biochemistry, University of Catania, Catania 95123, Italy; E-Mail: fgalvano@unict.it; 3 Biomedical Department of Internal and Specialist Medicine, University of Palermo, Palermo 90127, Italy; E-Mail: silvio.buscemi@unipa.it; 4 Department of Agri-Food and Environmental Systems and Management (DIGESA), University of Catania, Catania 95123, Italy; E-Mail: scuderia@unict.it; 5 Department of School Policies, Municipality of Catania, Catania 95131, Italy; E-Mail: margherita.matalone@comune.catania.it

**Keywords:** adolescents, Mediterranean diet, food intake, obesity, environment, nutrition transition

## Abstract

The present study aimed to examine the factors associated with increased Mediterranean diet (MD) adherence among a sample of Italian adolescents. A cross-sectional survey was conducted on 1135 students (13–16 years) attending 13 secondary schools of Sicily, southern Italy. Validated instruments were used for dietary assessment and the KIDMED score to assess adolescents’ adherence to the MD. A higher adherence to the MD was associated with high socioeconomic status (Odds Ratio [OR] 1.53, 95% Confidence Interval [CI]: 1.03–2.26) and high physical activity (OR 1.19, 95% CI: 1.02–1.70), whereas lower adherence was associated with living in an urban environment (OR 0.65, 95% CI: 0.44–0.97) and being obese (OR 0.59, 95% CI: 0.37–0.94). The adolescents’ KIDMED scores were inversely associated with adolescents’ intake of sweets, fast foods, fried foods, and sugary drinks, and directly with fruit, vegetables, pasta, fish, and cheese intakes. Urban-living adolescents were less likely to eat fruit and more prone to consume meat, sugary drinks, and fast food than rural-living adolescents. The latter were more likely to eat sweets and snacks. A general poor quality of food consumption in Italian adolescents away from the MD was reported, especially among those living in urban areas.

## 1. Introduction

Obesity among adolescents is one of the most important public health concern [1]. The prevalence of overweight among adolescents in the USA has increased rapidly over the past years [2] and similar trends have been recorded in European countries [3]. The most alarming rates have been reported especially for the Mediterranean region (Spain, Greece, Cyprus, and southern Italy) and the British islands, reaching the average of one third of the children [3]. Overweight and obesity among young are associated with severe metabolic consequences in youth that could persist during adulthood if not addressed early [4].

Numerous studies in adults reported the beneficial health effects of the Mediterranean dietary pattern, especially protection against cardiovascular diseases risk and certain cancers [5,6]. This dietary pattern is characterized by high consumption of vegetables fresh fruits, legumes, cereals, and a moderate intake of alcohol as main source of fiber and antioxidants, with fish, nuts, and olive oil as that ensure a high intake of monounsaturated fatty acids (MUFA), associated with low intake of trans fatty acids from meat and sweets [6]. Urbanization, connected with the phenomenon of nutrition transition and the shift from the traditional Mediterranean to a “Westernized” diet, has been hypothesized to explain the rise in the adolescence obesity precisely in those areas where it is supposed to be characterized by healthier dietary habits [4,7]. Indeed, demographic transition has been demonstrated to affect food choices in several countries of the Mediterranean area [8,9]. Moreover, modernization of society implies a series of unhealthy lifestyle habits such as sedentary activities (computer and television use) as well as less physical activity, which mostly affect young people [10].

Only few studies have been designed to explore the factors associated with the adherence to the Mediterranean diet specifically in the aforementioned areas, such as the Mediterranean islands [11,12] and even less interested young populations [13,14,15]. Some studies also emphasized the role of the environment (*i.e.*, rural and urban areas) and the role of socioeconomic status (SES) as factors significantly associated with the phenomenon of nutrition transition [16,17,18]. Studies assessing food habits are needed for planning population-based nutrition programs as well as nutrition education interventions. Furthermore, such public health efforts should be directed mainly towards children and adolescents to assess their nutrition habits and to make them establishing healthy eating habits that will have long lasting beneficial effects also in adult life.

Although Sicily is the biggest island of the Mediterranean area, data on food consumption and adherence to the Mediterranean diet are scarcely available [19,20,21], especially those regarding adolescents. Thus, the present study aimed to examine the factors associated with Mediterranean diet adherence in a sample of Italian adolescents living in Sicily, southern Italy.

## 2. Materials and Methods

### 2.1. Design and Setting

This cross-sectional study was based on 1135 adolescents of 13–16 years from 13 secondary schools of Sicily. The schools were chosen as representative of two urban or rural areas [22]. Ten schools were randomly selected in the urban area of the municipality of Catania. This was a stratified selection based on the socioeconomic level of the ten districts of the city to obtain a various range of SES among the participants. The classification of schools by SES was based on estimates of the district’s socioeconomic level in which schools were located: one school was selected from each district. For all enrolled school, all students attending last year of school from the urban area were invited to participate (*n* = 964). The schools in the rural areas were randomly chosen through a selection of provinces, which nutrition habits were supposed not to be influenced by geomorphologic characteristic (*i.e.*, lowland/mountainous area). Generally, one single school provided education for a large rural area of the island. Thus, three institutes in three rural provinces were selected and a total number of 210 students were invited to participate.

Data collection was performed between March and June 2011 by three trained medical doctors and a member of the Department of the school policies that followed a specific protocol to ensure that the same conditions were met for all students. Participants completed the questionnaire during school hours in the classroom in presence of a teacher and researchers encouraged participants to report any difficulties and questions raised during compilation of the questionnaire. A total of 1174 adolescents were invited to participate to the survey (excluding absent students from their class during the study) and 1135 (931 in the urban and 204 in the rural area) provided informed consent from parents and oral consent themself prior to filling out the questionnaire. Participation was not compulsory and participants were assured of complete anonymity. The study was approved by the ethics committee of the University of Catania and the Department of school policies of Catania.

### 2.2. Data Collection

The questionnaire consisted in a first part including demographic information such as the adolescents’ age, weight, height, their parent’s education level and job. SES definition was given by other surveys [14] taking into account education (primary, secondary/high school, and university) and occupation [unemployed and unskilled professions (*i.e.*, manual workers); partially skilled professions (*i.e.*, professors, nurses, *etc*.); skilled professions and white collars (*i.e.*, medical doctors, lawyers, managers, *etc*.)] of participants’ parents and was categorized in high, medium, and low according to the highest category achieved. Physical activity status was evaluated by frequency (times per week), duration (minutes per time), and intensity (expended calories) of sports-related and leisure time physical activity, and then categorized into: (i) low active (expended energy < 16.7 kJ (<4 kcal)/min, *i.e.*, walking slowly, cycling stationary, light stretching, *etc*.); (ii) moderately active (expended energy 16.7–29.3 kJ (4–7 kcal)/min, *i.e.*, walking briskly, cycling outdoors, swimming moderate effort, *etc*.); and (iii) highly active (expended energy > 29.3 kJ (>7 kcal)/min, *i.e.*, walking briskly uphill, long-distance running, cycling fast or racing, swimming fast crawl, *etc*.). BMI was computed as weight in kilograms divided by the square of height in meters, and international age- and gender-specific cut-off points for children according to the International Obesity Task Force were used to define their weight status in terms of underweight, overweight and obesity [23].

### 2.3. Dietary Intake Measurements

The second part of the questionnaire focused on the dietary assessment based on a revised version of several food-frequency questionnaires (FFQs) developed for Italian population [24,25,26] and previously validated [27], asking about the consumption frequency of important sources of dietary fibre (fruit, vegetables, breakfast cereals, bread), calcium (milk, cheese, yogurt) and items typically of the youth food culture (fast foods, chips, sweets or chocolates, carbonated sugared soft drinks and diet soft drinks). Since the instrument was developed for the northern Italian population, slight modifications on specific foods (*i.e.*, inclusion of specific fast foods and sweets) consumed more likely in the south rather than in the north of Italy, were done. The food list included 116 items grouped in 11 principal food categories. The response options for each food item were categorized in 10 categories: “seldom/never”, “2–3 times/month”, “once a week”, “2–3 times/week”, “4–6 times/week”, “once a day”, “2–3 times/day”, “4–5 times/day”, “6 or more times/day”. The average portion sizes were identified in terms of household measures, pack sizes and units. For the analyses, the items of the FFQ containing more food components were separated according to their ingredients. The monthly food consumption was calculated in gr (or mL) and then converted in 24-h intake. Energy density and nutrient intakes were calculated by using a web program containing the food composition tables of the European Institute of Oncology database [28]. 

The KIDMED index (Mediterranean Diet Quality Index for children and adolescent) developed by Serra-Majem *et al*. [29] was used to evaluate the different adherence to the Mediterranean diet by measuring the consumption of 16 components: questions denoting a negative connotation with respect to the Mediterranean diet (*i.e.*, fast foods, baked goods, sweets, *etc*.) were assigned a value of −1, and those with a positive aspect (*i.e.*, fruit, vegetables, cereals, nuts, *etc*.) were scored +1. According to the KIDMED index, a score of 0–3 reflects a poor adherence to the Mediterranean diet, a score of 4–7 describes average adherence, and a score of 8–12 a good adherence.

### 2.4. Statistical Analysis

Categorical variables are presented as absolute frequencies and percentages, whereas continuous variables are presented as means ± standard deviations (SDs). Differences between categorical variables were tested by Chi-square test. Normality of variables’ distribution was tested by Kolmogorov-Smirnov test. Associations between normal distributed variables were tested by Student’s independent *t*-test, whereas Mann-Whitney *U*-test was used for not-normally distributed continuous variables. Respectively, one-way ANOVA and Kruskall-Wallis were applied for multiple comparisons.

Multivariate logistic regression analyses were performed in order to estimate the Odds Ratios (ORs) and the corresponding 95% Confidence Intervals (CIs) of higher adherence to the Mediterranean diet (namely highest tertile of the score), adjusting for all other variables as potential confounders. Linear regression analyses were used to evaluate the association of micro-, macro-nutrients, and food groups intake with the KIDMED score. Finally, multivariate logistic regression analyses were then applied to place of residence (urban *vs*. rural), BMI status (normal *vs*. overweight/obese), and SES level (low *vs*. medium/high) as the dependent variables to estimate the association between the aforementioned variables and food groups intake. For the sake of simplicity in the presentation of the data and in order to get clearer and more meaningful ORs and 95% CI, consumption of food groups are presented in 10 g/day.

All reported *p*-values were based on two-sided tests and compared to a significance level of 5%. SPSS 17.0 software (Statistical Package for Social Sciences, Chicago, IL, USA) was used for all statistical analyses.

## 3. Results

The mean age of the adolescents was 13 ± 0.5 with no significant differences on any variable of interest. Nearly half of participants were overweight or obese with a mean BMI of 21.8 ± 3.1. The characteristics of the 1135 adolescents by gender are presented in Table 1. Male adolescents were significantly more obese but also more active than females. The characteristics of the participants have been also explored by level of adherence to the Mediterranean diet (namely mean values and tertiles of KIDMED score) (Table 2). An increased adherence to the Mediterranean diet was associated with high SES (OR 1.53, 95% CI: 1.03–2.26) and high physical activity (OR 1.19, 95% CI: 1.02–1.70), whereas poor adherence was associated with living in the urban environment (OR 0.65, 95% CI: 0.44–0.97) and being obese (OR 0.59, 95% CI: 0.37–0.94).

Adolescents’ daily energy, nutrients, and food groups intake by gender revealed no substantial differences between male and female except those due to the greater amount of food consumed by males (Table 3). When analyzing the same variables by tertiles of KIDMED scores, a significant inverse correlation between the KIDMED score and saturated fatty acids (β = −0.099, *p* < 0.001) and a direct correlation with carbohydrates (β = 0.004, *p* < 0.001) and fibers (β = 0.057, *p* < 0.001) were found (Table 4). Among the food groups, consumption of sweets (β = −0.008, *p* < 0.001), fast foods (β = −0.009, *p* < 0.001), fried potatoes (chips) (β = −0.007, *p* < 0.05), and sugary drinks (β = −0.003, *p* < 0.001), were inversely correlated with the KIDMED score, whereas a direct correlation with fruit (β = 0.010, *p* < 0.001), vegetables (β = 0.011 *p* < 0.001), pasta (β = 0.019, *p* < 0.001), fish (β = 0.029, *p* < 0.001), and cheese (β = 0.021, *p* < 0.001) was found (Table 4).

Table 5 shows the distribution of the daily amount of food group intake by place of living (*i.e.*, rural *vs*. urban), BMI status (normal *vs*. overweight/obese) and SES (low *vs*. medium/high). Urban adolescents were less likely to eat fruit (OR 0.96, 95% CI: 0.94–0.98) but also less sweets (OR 0.94, 95% CI: 0.90–0.98) and snacks (OR 0.89, 95% CI: 0.83–0.94) than rural adolescents. On the other hand, urban adolescents were reporting to consume more meat (OR 1.09, 95% CI: 1.03–1.17), cheese (OR 1.20, 95% CI: 1.01–1.20), fast foods (OR 1.04, 95% CI: 1.01–1.08), and sugary drinks (OR 1.05, 95% CI: 1.03–1.08). Despite food consumption by BMI and SES evidenced several differences in the univariate models (data not shown), after adjusting for confounders (place of living, BMI category, SES level) and for demographic covariates (age and gender), only few of those differences were still statistically significant (Table 5). Particularly, unhealthy eating behaviors were found in overweight and obese adolescents, resulting to eat more sweets (OR 1.05, 95% CI: 1.01–1.08) and snacks (OR 1.05, 95% CI: 1.03–1.08) than normal weighted, whereas participants with medium and high SES were less likely to consume sugary drinks (OR 0.98, 95% CI: 0.96–0.99).

**Table 1:** Socio-demographic and lifestyle characteristics of the 1135 participants, by gender and place of living.

	Total			Male		Female		*p* ^a^	Rural		Urban		*p* ^a^
	*n*	(%)		*n*	(%)	*n*	(%)		*n*	(%)	*n*	(%)	
Gender													0.720
Male	627	(55.0)		-		-			115	(56.4)	512	(55.0)	
Female	508	(45.0)		-		-			89	(43.6)	419	45.0	
BMI								<0.001					<0.001
Under/normal weight	612	(53.7)		286	(45.6)	326	(64.2)		139	(68.1)	473	(50.8)	
Overweight	411	(36.1)		254	(40.5)	157	(30.9)		54	(26.5)	357	(38.3)	
Obese	112	(9.8)		87	(13.9)	25	(4.9)		11	(5.4)	101	(10.8)	
Socio-economic status								0.671					0.004
Low	496	(43.5)		271	(43.2)	225	(44.3)		109	(53.4)	387	(41.6)	
Medium	434	(38.1)		237	(37.8)	197	(38.8)		70	(34.3)	364	(39.1)	
High	205	(18.0)		119	(19.0)	86	(16.9)		25	(12.3)	180	(19.3)	
Physical activity level								<0.001					<0.001
Low	510	(44.9)		240	(38.3)	270	(53.1)		49	(24.0)	461	(49.5)	
Moderate	387	(34.1)		232	(37.0)	155	(30.5)		92	(45.1)	295	(31.7)	
High	238	(21.0)		155	(24.7)	83	(16.3)		63	(30.9)	175	(18.8)	
Place of living								0.720					
Rural	204	(17.9)		115	(18.3)	89	(17.5)		-		-		
Urban	931	(81.7)		512	(81.7)	419	(82.5)		-		-		

^a^ significance by Chi-square test.

**Table 2:** Socio-demographic and lifestyle characteristics of the study population, by tertiles of KIDMED diet adherence scores, mean KIDMED scores, and relative odd ratios by multiple logistic regression analysis of factors associated with high adherence to the Mediterranean diet, adjusted for all covariates.

	KIDMED score		*p* ^a^	KIDMED score low			KIDMED score medium			KIDMED score high		*p* ^b^	Odds of high adherence ^c^
	Mean	(SD)		*n*	(%)		*n*	(%)		*n*	(%)		OR (95% CI)
Gender			0.393									0.434	
Male	4.9	(2.3)		167	(57.6)		398	(53.9)		62	(58.5)		1
Female	5.0	(2.1)		123	(42.4)		341	(46.1)		44	(41.5)		1.06 (0.79–1.41)
BMI			<0.001									<0.001	
Under/normal weight	5.4	(2.1)		120	(41.4)		418	(56.6)		74	(69.8)		1
Overweight	4.5	(2.2)		135	(46.6)		249	(33.7)		27	(25.5)		0.52 (0.39–0.70)
Obese	4.3	(1.9)		35	(12.1)		72	(9.7)		5	(4.7)		0.59 (0.37–0.94)
Socio-economic status			0.015									0.039	
Low	4.8	(2.2)		141	(48.6)		316	(42.8)		39	(36.8)		1
Medium	5.0	(2.2)		105	(36.2)		291	(39.4)		38	(35.8)		1.27 (0.94–1.71)
High	5.3	(2.2)		44	(15.2)		132	(17.9)		29	(27.4)		1.53 (1.03–2.26)
Physical activity level			0.039									0.044	
Low	4.8	(2.2)		144	(49.7)		322	(43.6)		24	(22.7)		1
Moderate	5.0	(2.1)		90	(31.0)		262	(35.5)		35	(33.0)		1.17 (0.86–1.23)
High	5.2	(2.2)		56	(19.3)		155	(21.0)		47	(44.3)		1.19 (1.02–1.70)
Place of living			<0.001									0.020	
Rural	5.8	(1.8)		37	(12.8)		143	(19.4)		24	(22.6)		1
Urban	4.8	(2.2)		253	(87.2)		596	(80.6)		82	(77.4)		0.65 (0.44–0.97)

^a^ significance by Student’s *t*-test (or Mann-Whitney *U*-test) or ANOVA (or Kruskall-Wallis), as appropriate; ^b^ significance by Chi-square test; ^c^ multivariate logistic regression model with KIDMED (low *vs*. medium-high tertile) as dependent variable; OR, odds ratio; CI, confidence interval.

**Table 3:** Daily energy, nutrients, and food groups intake of the study population, overall and stratified by gender (*n* = 1135).

	All *N* = 1135			Male *N* = 627			Female *N* = 508			*p* ^a^
	Mean	(SD)		Mean	(SD)		Mean	(SD)		
Energy intake (kcal)	2274	(246)		2337	(238)		2198	(233)		<0.001
Energy intake (kJ)	9782	(1057)		10049	(1023)		9453	(1004)		<0.001
Macronutrients										
Proteins (g)	57.9	(13.6)		62.3	(14.5)		52.4	(9.9)		<0.001
Carbohydrates (g)	309.0	(105.9)		327.9	(102.4)		285.7	(105.5)		<0.001
Total sugar (g)	133.8	(16.0)		146.4	(9.6)		118.2	(4.4)		<0.001
Fat (g)	94.7	(9.4)		96.7	(9.3)		92.2	(8.9)		<0.001
SFA (g)	31.8	(3.7)		33.7	(3.2)		29.3	(2.7)		<0.001
MUFA (g)	26.9	(3.2)		27.8	(3.1)		25.8	(3.1)		<0.001
PUFA (g)	10.4	(1.5)		11.0	(1.4)		9.6	(1.1)		<0.001
Micronutrients										
Cholesterol (mg)	236.1	(26.1)		237.4	(26.3)		234.4	(25.8)		0.060
Fiber (g)	33.4	(3.7)		35.4	(3.2)		31.0	(2.6)		<0.001
Vitamin C (mg)	136.5	(14.8)		135.4	(14.7)		137.8	(14.9)		0.007
Vitamin A (μg)	581.2	(71.3)		577.4	(70.5)		586.0	(72.1)		0.044
Vitamin B12 (µg)	2.4	(1.0)		2.4	(1.0)		2.3	(1.01)		0.178
Folate (µg)	271.0	(28.9)		270.9	(29.0)		271.2	(28.8)		0.886
Iron (mg)	7.2	(0.8)		7.2	(0.8)		7.2	(0.8)		0.144
Zinc (mg)	6.5	(0.5)		6.5	(0.5)		6.4	(0.5)		0.176
Calcium (mg)	922.6	(72.9)		920.5	(73.5)		925.2	(72.2)		0.280
Food groups										
Pasta/rice (g)	58.5	(26.5)		57.9	(26.5)		59.1	(26.5)		0.552
Meat (g)	37.8	(27.4)		37.0	(27.1)		38.8	(27.7)		0.412
Fish (g)	13.5	(17.8)		14.3	(18.6)		12.4	(16.8)		0.060
Cheese (g)	19.2	(19.3)		19.1	(19.2)		19.2	(19.3)		0.851
Eggs (g)	9.6	(13.4)		9.6	(13.3)		9.6	(13.5)		0.857
Vegetables (g)	42.5	(62.9)		40.4	(62.2)		45.1	(63.7)		0.214
Fruit (g)	56.1	(65.7)		55.2	(66.6)		57.3	(64.7)		0.592
Sweets (g)	27.1	(38.7)		26.1	(35.5)		28.5	(42.2)		0.233
Fast foods (g)	44.8	(56.1)		47.5	(57.1)		41.4	(54.8)		0.072
Snacks (g)	18.9	(24.3)		19.2	(24.2)		18.5	(24.5)		0.623
Sugary Drinks (mL)	58.3	(85.0)		60.1	(85.0)		56.1	(85.1)		0.450

^a^ significance by Student’s *t*-test (or Mann-Whitney *U*-test) or ANOVA (or Kruskall-Wallis), as appropriate.

**Table 4:** Daily energy, nutrients, and food groups intake of the study population, by tertiles of KIDMED diet adherence scores, and linear regression analysis with the KIDMED score.

	KIDMED score low			KIDMED score medium			KIDMED score high			*p* ^a^		β ^b^	
	Mean	(SD)		Mean	(SD)		Mean	(SD)					
Energy intake (kcal)	2246.1	(252.2)		2291.3	(240.7)		2239.2	(254.1)		0.009		0.000	
Energy intake (kJ)	9658.4	(1084.5)		9852.7	(1035.0)		9628.8	(1092.7)		0.009		0.000	
Macronutrients													
Proteins (g)	57.8	(13.7)		57.8	(13.2)		58.2	(15.9)		0.973		−0.009	
Carbohydrates (g)	284.1	(111.6)		311.5	(104.4)		306.0	(76.6)		<0.001		0.004	**
Total sugar (g)	132.8	(15.5)		134.9	(16.2)		128.8	(15.4)		0.001		−0.004	
Fat (g)	94.3	(9.4)		94.8	(9.4)		94.9	(9.4)		0.726		0.003	
SFA (g)	33.3	(3.4)		31.1	(3.6)		32.1	(3.8)		<0.001		−0.099	**
MUFA (g)	27.0	(3.3)		26.9	(3.2)		26.8	(3.3)		0.702		−0.013	
PUFA (g)	10.4	(1.5)		10.4	(1.4)		10.4	(1.6)		0.030		−0.025	
Micronutrients													
Cholesterol (mg)	233.7	(25.7)		236.8	(26.1)		237.6	(26.9)		0.187		0.003	
Fiber (g)	33.3	(3.4)		33.1	(3.6)		36.1	(3.8)		<0.001		0.057	*
Vitamin C (mg)	135.8	(15.1)		136.8	(14.9)		136.2	(13.6)		0.590		0.004	
Vitamin A (μg)	580.0	(69.6)		583.0	(71.6)		572.2	(73.6)		0.324		−0.001	
Vitamin B12 (μg)	2.4	(1.0)		2.4	(1.0)		2.4	(1.0)		0.509		−0.084	
Folate (µg)	270.3	(28.9)		270.9	(28.6)		273.7	(31.0)		0.580		−0.001	
Iron (mg)	7.2	(0.8)		7.2	(0.8)		7.2	(0.8)		0.572		0.031	
Zinc(mg)	6.5	(0.5)		6.5	(0.5)		6.5	(0.5)		0.522		0.009	
Calcium (mg)	927.0	(72.7)		921.3	(73.4)		919.3	(70.5)		0.475		−0.001	
Food groups													
Pasta/rice (g)	50.8	(29.2)		59.6	(25.8)		71.6	(15.3)		<0.001		0.019	**
Meat (g)	37.5	(28.0)		37.2	(27.3)		43.3	(25.8)		0.095		0.003	
Fish (g)	9.0	(12.7)		13.7	(18.0)		23.7	(23.4)		<0.001		0.029	**
Cheese (g)	14.7	(17.0)		20.0	(19.4)		26.1	(21.0)		<0.001		0.021	**
Eggs (g)	8.7	(12.5)		9.9	(13.8)		9.9	(12.5)		0.407		0.008	
Vegetables (g)	15.5	(37.4)		48.7	(65.2)		73.5	(75.9)		<0.001		0.011	**
Fruit (g)	28.5	(50.8)		62.4	(65.9)		88.2	(75.3)		<0.001		0.010	**
Sweets (g)	34.1	(42.7)		25.8	(37.8)		17.6	(29.1)		<0.001		−0.008	**
Fast foods (g)	58.1	(61.9)		43.2	(55.4)		19.1	(27.1)		<0.001		−0.009	**
Snacks (g)	21.9	(26.1)		18.5	(23.6)		13.9	(23.5)		0.011		−0.007	*
Sugary Drinks (mL)	67.2	(87.6)		58.2	(85.7)		34.9	(67.3)		0.004		−0.003	**

^a^ significance by ANOVA or Kruskall-Wallis test, as appropriate; ^b^ adjusted also for age, gender, BMI, physical activity level, socioeconomic status and place of living; * *p* < 0.05; ** *p* < 0.001.

**Table 5:** Consumption (10 g/day) of food groups of the study population by place of living (rural *vs*. urban), BMI status (normal *vs*. overweight/obese), and socioeconomic status (low *vs*. medium/high) and relative odd ratiosby multiple logistic regression analysis (first category as reference.

	Urban	Rural	*p* ^a^	Urban *vs*. rural ^b^		Normal	Overweight/obese	*P* ^a^	Normal *vs*. overweight/obese ^c^		Low	Medium/high SES	*p* ^a^	Low *vs*. medium/high SES ^d^
	Mean (SD)	Mean (SD)		OR (95% CI)		Mean (SD)	Mean (SD)		OR (95% CI)		Mean (SD)	Mean (SD)		OR (95% CI)
Pasta/rice	6.0 (2)	5.7 (2.7)	0.131	0.94 (0.88–1.01)		5.8 (2.6)	5.8 (2.7)	0.995	1.01 (0.96–1.05)		5.9 (2.6)	5.7 (2.7)	0.399	0.97 (0.93–1.02)
Meat	3.4 (2.5)	3.8 (2.7)	0.032	1.09 (1.03–1.17)		3.7 (2.7)	3.8 (2.7)	0.384	1.03 (0.98–1.07)		3.6 (2.7)	3.8 (2.7)	0.331	1.02 (0.97–1.06)
Fish	1.5 (1.6)	1.3 (1.8)	0.046	0.92 (0.84–1.01)		1.3 (1.7)	1.3 (1.8)	0.953	1.01 (0.94–1.08)		1.4 (1.8)	1.3 (1.7)	0.382	0.99 (0.92–1.06)
Cheese	1.8 (1.7)	2.0 (2.0)	0.225	1.10 (1.01–1.20)		2.0 (2.0)	1.9 (2.0)	0.494	0.98 (0.92–1.04)		1.9 (2.0)	2.0 (2.0)	0.679	1.01 (0.95–1.08)
Eggs	1.3 (1.2)	1.0 (1.4)	0.020	0.90 (0.81–1.01)		1.2 (1.4)	1.0 (1.4)	0.062	0.93 (0.85–1.02)		1.1 (1.5)	1.0 (1.3)	0.193	0.95 (0.87–1.04)
Vegetables	4.9 (4.9)	4.1 (6.5)	0.121	0.98 (0.96–1.01)		4.6 (6.4)	3.8 (6.0)	0.043	0.99 (0.97–1.01)		4.0 (6.0)	4.5 (6.4)	0.180	1.01 (0.99–1.03)
Fruit	7.2 (5.7)	5.2 (6.6)	<0.001	0.96 (0.94–0.98)		6.0 (6.6)	5.1 (6.3)	0.011	0.99 (0.97–1.01)		5.2 (6.1)	5.9 (6.8)	0.100	1.01 (0.99–1.03)
Sweets	3.3 (2.9)	2.7 (4.0)	0.038	0.94 (0.90–0.98)		2.5 (3.8)	3.2 (3.9)	0.003	1.05 (1.01–1.08)		3.0 (4.0)	2.6 (3.7)	0.087	0.99 (0.96–1.03)
Fast foods	3.8 (3.1)	4.7 (6.1)	0.030	1.04 (1.01–1.08)		3.7 (5.2)	5.5 (6.1)	<0.001	1.05 (1.03–1.08)		4.8 (5.9)	4.3 (5.6)	0.167	0.99 (0.97–1.02)
Snacks	2.4 (2.7)	1.7 (2.5)	0.001	0.89 (0.83–0.94)		1.8 (2.5)	1.8 (2.5)	0.766	0.96 (0.91–1.01)		2.0 (2.5)	1.7 (2.5)	0.029	0.98 (0.93–1.03)
Sugary drinks	6.2 (9.1)	3.9 (4.6)	<0.001	1.05 (1.03–1.08)		5.2 (7.9)	6.5 (9.1)	0.012	1.01 (0.99–1.02)		6.9 (9.0)	5.0 (8.0)	<0.001	0.98 (0.96–0.99)

^a^ significance by Student’s *t*-test (or Mann-Whitney *U*-test) or ANOVA (or Kruskall-Wallis), as appropriate; ^b^ adjusted for age, gender, BMI, physical activity level and socioeconomic status (SES); ^c^ adjusted for age, gender, place of living, physical activity level and SES; ^d^ adjusted for age, gender, place of living, physical activity level and BMI.

## 4. Discussion

We reported the nutritional data from 1135 adolescents living in Sicily, southern Italy, and evaluated the factors associated with their adherence to the Mediterranean diet. Adolescents living in the urban areas of the island were more likely to adopt dietary behaviors different than the traditional Mediterranean dietary pattern. Moreover, those away from the Mediterranean diet, reported a high intake of unhealthy food groups, such as fast foods, and a decreased intake of fruit.

The studies reporting data regarding the dietary patterns of adolescents living in the Mediterranean area are scarce [30]. Moreover, studies which compare dietary habits of young populations by place of living in the Mediterranean islands are even less [13,14,15]. Regarding the Italian population, a recent study conducted in North Italy explored the dietary habits between 927 eight-year-old, rural and urban children [31], but none has been conducted in the Italian islands where it is supposed to be a specific dietary pattern closer to the principles of the Mediterranean diet compared with the northern Italian areas [32]. At our knowledge, this work gives an important contribution to the scientific literature since no previous studies have reported specific eating habits in two representative samples of urban and rural areas of Sicily.

We used the KIDMED index to assess the adherence of the whole diet of the adolescents to the Mediterranean diet model. Results appeared to be in line with the magnitude of the differences reported in frequency of consumption of all major food categories, being adolescents living in rural areas more likely to adhere to a Mediterranean diet even after adjusting for potential confounders [33]. The level of adherence to the Mediterranean diet was generally low. Indeed, only 11% of adolescents living in rural areas and 9% of those living in urban, reported to have a good adherence (namely reaching the highest tertile of the index) to the traditional dietary pattern. These rates are even lower than those previously reported for Cypriot, Balearic and Greek adolescents [13,14,34]. It has been showed that adherence was strongly influenced by SES of parents of participants, and similar associations were found also in adult population [17]. It is debatable if this association depends on increased economic possibilities of family with high incomes, or rather high SES represent a surrogate of higher cultural level, which may lead to increased knowledge on health topic and, thus, on healthier eating behaviors. However, in our study reported a general better quality of the diet among those adolescents more adherent to the Mediterranean diet, since they consumed smaller quantities of sweets, fast foods, sugary drinks, and chips, and reported higher intakes of fruit, vegetables, and fish, compared with lower adherent. As well, also BMI resulted independently associated to a higher adherence to the Mediterranean dietary pattern, as reported in previous studies [13]. Physically active adolescents showed higher adherence and higher odds of adherence to the Mediterranean dietary pattern than low active and sedentary adolescents. Since higher adherence to the Mediterranean diet were found in rural areas, it may be possible that rural adolescents are more likely to spend time in high energy-expenditure activities than urban counterpart. However, as reported elsewhere [13], healthy lifestyle variables tend to cluster, including both eating and lifestyle behaviors that may result protective against different diseases.

Nutrition analysis indicated that rural and urban adolescents consume equivalent amount of pasta, fish, eggs, and vegetables. Participants living in the rural area were more likely to consume higher quantities of fruit than those living in the urban that, on the contrary, were inclined to eat more meat, cheese, snacks, and sugary drinks. Despite the fact that health benefits of fruit and vegetable intake have been corroborated by numerous experimental studies [35,36], several surveys conducted in both adult and young population in the European contest revealed that younger age groups showed the most compromised intake of fruits and vegetables compared with adults [37,38]. The stratified analysis by place of living revealed a stronger association of fruit consumption with the rural environment rather than with BMI. In general, environmental factors appeared to be important for adolescents’ fruit intake and was indicative of a higher adherence to the Mediterranean diet when compared with adolescents living in the urban environment. Curiously, we also found some contrasting data, in that rural adolescents were more like to consume sweets compared with urban ones. A possible explanation of such consumption could be identified in the intrinsic differences of rural and urban society and family in the contest of a Mediterranean island. Occupational status of mothers living in the rural areas is generally low, being the most of them not working, as assessed in a previous study [26]. The role of mother in the family contest is crucial in determining quantity and quality of children nutrition, given a higher percentage of mothers from rural areas cooking every day for their family compared with urban families [14]. Thus, the higher amount of time spent in home and a “cultural” addiction in providing their families with home-made food, may lead to a higher consumption of traditional desserts rather than bakeries and confectioneries. On the other hand, the Sicilian cuisine is rich also in fried courses, thus adolescents in the rural areas might be more exposed to such kind of nutrition. Such eating preferences have been underlined in other studies [39].

Despite research conducted on adolescents’ food habits living in the Mediterranean area is weak, few evidences have been found with a certain grade of disagree. Some studies reported complete similarity of dietary habits of children and adolescents with several common elements in food culture of those living in Sicily [15,33]. Some older studies showed minor or no substantial differences regarding diet composition of rural and urban children [40,41]. We hypothesize that the progressive “Westernization” of the diet is progressively involving also more rural or deprived areas, making the differences between rural and urban areas disappear. It may be possible that few differences still exist but mostly regard some traditional and conservative environments, such the islander context. Contrarily to other studies [42,43], the results regarding the obesity rates among rural and urban adolescents were significant, suggesting that an imbalance on energy intake and expenditure does exist for urban and rural adolescents. As conclusion of such results, an association between adherence to the Mediterranean diet, BMI, and place of living seems to exist, although a clear cause-effect relation can’t be identified. One could speculate that people living in the rural environment place may be influenced in their diet quality with a higher adherence to the Mediterranean diet, leading to be less likely to be obese [44]. However, existing studies are conducted mostly on adults and prospective studies conducted on children and adolescents are needed to detect and underline such associations.

Some limitations of the present study need to be addressed. First, it was a cross-sectional study design. Therefore, conclusions cannot be attributed to plausible causes, but instead they are valuable indications that can be used in future investigations. Moreover, data collected were self-reported. This may lead to misreporting and recall bias because of the nature of the study and the young age of participants. Although such measure is commonly used in scientific literature and has demonstrated acceptable reliability and validity, it is difficult to quantify and categorize certain variables (*i.e.*, physical activity) on the basis of self-reported questionnaires. Second, although the instrument has been previously well validated, we only collected adolescents’ information but more valid data from multiple sources (*i.e.*, parents and adolescents) may reinforce the findings of nutrition studies conducted on young participants. Finally, dietary information was collected through the questionnaire, but we were not able to detect the exact portion size or amount consumed of each foods through other instruments, such as a 24-h recall. Thus, the estimation of nutrients depended only on the FFQ, which may have led to lack of accuracy.

## 5. Conclusions

This study provides important information regarding a specific population of Italian adolescents living in Sicily. Children and adolescents represent priority targets for action and understanding their dietary habits is essential to plan educational interventions aimed at improving eating behaviours and, consequently, their health. However, differences in adherence to the Mediterranean diet and foods intake between urban and rural adolescents should be taken in account. Strategies specifically targeting urban adolescents may need to focus on specific aspects, such as information programs on importance of fruit and vegetables consumption, whereas educational campaigns should be conducted to improve food quality, with special regards on fat or calorie dense foods in both environments. Finally, also families should be targeted by intervention programs, persuading parents to make healthier choices at the time of food purchase and preparation at home.
